# Clinical performance of a novel brief erectile dysfunction scale and comparison with the international index of erectile function-5

**DOI:** 10.3389/fpubh.2026.1901986

**Published:** 2026-08-13

**Authors:** Chengxu Cao, Jian Wang, Zhanghu Li, Bin Cai, Ziyue Zong, Yang Sun, Yun Chen

**Affiliations:** 1 Department of Andrology, Affiliated Hospital of Nanjing University of Chinese Medicine/Jiangsu Provincial Hospital of Chinese Medicine, Nanjing, Jiangsu, China; 2 Department of Andrology, Yangzhou Hospital of Traditional Chinese Medicine, Yangzhou, Jiangsu, China

**Keywords:** brief scale, clinical study, erectile dysfunction, international index of erectile function-5, rapid screening tool

## Abstract

**Objective:**

This study aimed to develop a novel brief erectile dysfunction (ED) scale to serve as a rapid clinical tool for grading ED severity, thereby providing a more concise and efficient diagnostic instrument for routine practice.

**Methods:**

A total of 528 eligible patients were enrolled in this study. Each participant independently completed both the novel brief scale and the International Index of Erectile Function-5 (IIEF-5), and subsequently underwent RigiScan monitoring for nocturnal penile tumescence and rigidity (NPTR), intracavernosal injection (ICI) of vasoactive agents, a comprehensive battery of psychological assessments—including the Symptom Checklist-90 (SCL-90), the Eysenck Personality Questionnaire (EPQ), the Hamilton Depression Rating Scale (HAMD), the Hamilton Anxiety Rating Scale (HAMA), and the State-Trait Anxiety Inventory (STAI)—as well as a panel of laboratory tests. The results from all these examinations were compared with the scores obtained from both the brief scale and the IIEF-5 to evaluate the diagnostic performance of the novel instrument. Weighted Cohen's kappa coefficients were calculated to assess the concordance between the two scales in terms of severity classification, and receiver operating characteristic (ROC) curve analysis was performed using RigiScan grading as the reference standard, with the area under the curve (AUC), sensitivity, specificity, and other diagnostic metrics being computed.

**Results:**

The brief scale exhibited a strong correlation with the IIEF-5 (Spearman's ^*^r^*^ = 0.77), with a weighted Cohen's kappa of 0.773 (95% CI: 0.725–0.817). Both scales showed highly consistent associations with RigiScan parameters, ICI outcomes, scores on the various psychological inventories, and laboratory measurements. Moreover, the two instruments demonstrated comparable consistency in capturing treatment-related changes in erectile function. Using RigiScan grading as the reference standard, the brief scale yielded an area under the curve (AUC) of 0.77 (95% CI: 0.71–0.83) for the diagnosis of ED, with a sensitivity of 77.8%, a specificity of 78.1%, a false-positive rate of 21.9%, and a false-negative rate of 22.2%.

**Conclusions:**

The novel brief scale may be clinically applicable for assessing ED across a spectrum of severity. It affords the advantages of greater speed and convenience and holds promise as a complementary rapid screening tool for the preliminary assessment of ED.

## 1 Introduction

Erectile dysfunction (ED) is defined as the persistent inability to attain or maintain an erection of sufficient rigidity for satisfactory sexual intercourse. Kitaw et al. (1) reported that approximately 322 million men worldwide were living with ED in 2025. The condition has become a major public health concern, substantially compromising the quality of life, interpersonal relationships, and psychological wellbeing of both patients and their partners (2).

ED is currently evaluated using two principal approaches: objective diagnostic tests and patient-reported outcome measures. Nocturnal penile tumescence and rigidity (NPTR) is widely employed to differentiate psychogenic from organic ED (3). Intracavernosal injection (ICI) involves the injection of vasoactive agents into the corpora cavernosa to induce an erection, thereby providing an initial assessment of vascular function (4). Given the well-established link between ED and various systemic disorders, a panel of laboratory parameters—including alanine aminotransferase (ALT), aspartate aminotransferase (AST) (5), fasting blood glucose (6), serum uric acid (7), lipid profile (5), and sex hormones (8)—provides biological validity for adjunctive diagnosis. Psychogenic ED accounts for a considerable proportion of cases among younger men (9, 10), and several psychological instruments are used to evaluate ED-related mental states, including the Hamilton Anxiety Rating Scale (HAMA), the Hamilton Depression Rating Scale (HAMD), the Eysenck Personality Questionnaire (EPQ) (11), the Symptom Checklist-90 (SCL-90) (12), and the State–Trait Anxiety Inventory (STAI). Commonly adopted self-report scales include the International Index of Erectile Function (IIEF), its abridged version—the IIEF-5, and the Erection Quality Scale (EQS). The IIEF has been widely recommended as a primary endpoint and severity-grading tool in ED clinical trials (13). The diagnostic performance of the IIEF-5 has also been substantiated: a 2007 study reported a sensitivity of 94.7%−98% and a specificity of 88%−95.2% for diagnosing ED (14), and a 2020 study confirmed that the electronic version of the IIEF-5 demonstrates reliability and validity highly consistent with the paper-based version (15).

Nevertheless, a growing body of evidence has brought to light several limitations of the IIEF-5 in clinical practice. First, the scale implicitly assumes that all items tap a single “erectile function” dimension, whereas the full-length IIEF encompasses five distinct domains. A 2019 systematic review concluded that establishing the unidimensionality of the IIEF-5 is an urgent research priority (16). Second, the IIEF-5 requires patients to recall sexual activity over the preceding 6 months. Owing to the lengthy recall period, patients may experience considerable difficulty in accurately recollecting their responses for each individual item, which can readily lead to invalid scoring of key items and consequently elevate the risk of missing data. Third, diagnostic cut-off values vary across cultural settings. The optimal cut-off for the Malay version of the IIEF-5 yielded a sensitivity of 85% and a specificity of 75%, figures markedly lower than those originally reported in Western populations (17). Fourth, the IIEF-5 cannot discriminate between different ED etiologies, and scores from patients with distinct underlying causes overlap considerably. When pre-mature ejaculation (PE) co-occurs with ED, item 5 is particularly prone to generating false-positive diagnoses (18). Finally, completing the IIEF-5 typically takes 3–5 min. Nevertheless, in busy outpatient settings, the additional time required for instruction and explanation still poses a practical burden (13, 15).

Against this background, the present study developed a brief, three-item scale grounded in the definition of ED and clinical practice, specifically designed to rapidly grade ED severity. The scale can be completed in an average of 1–2 min.

## 2 Materials and methods

### 2.1 Scale development

**Figure 1:**
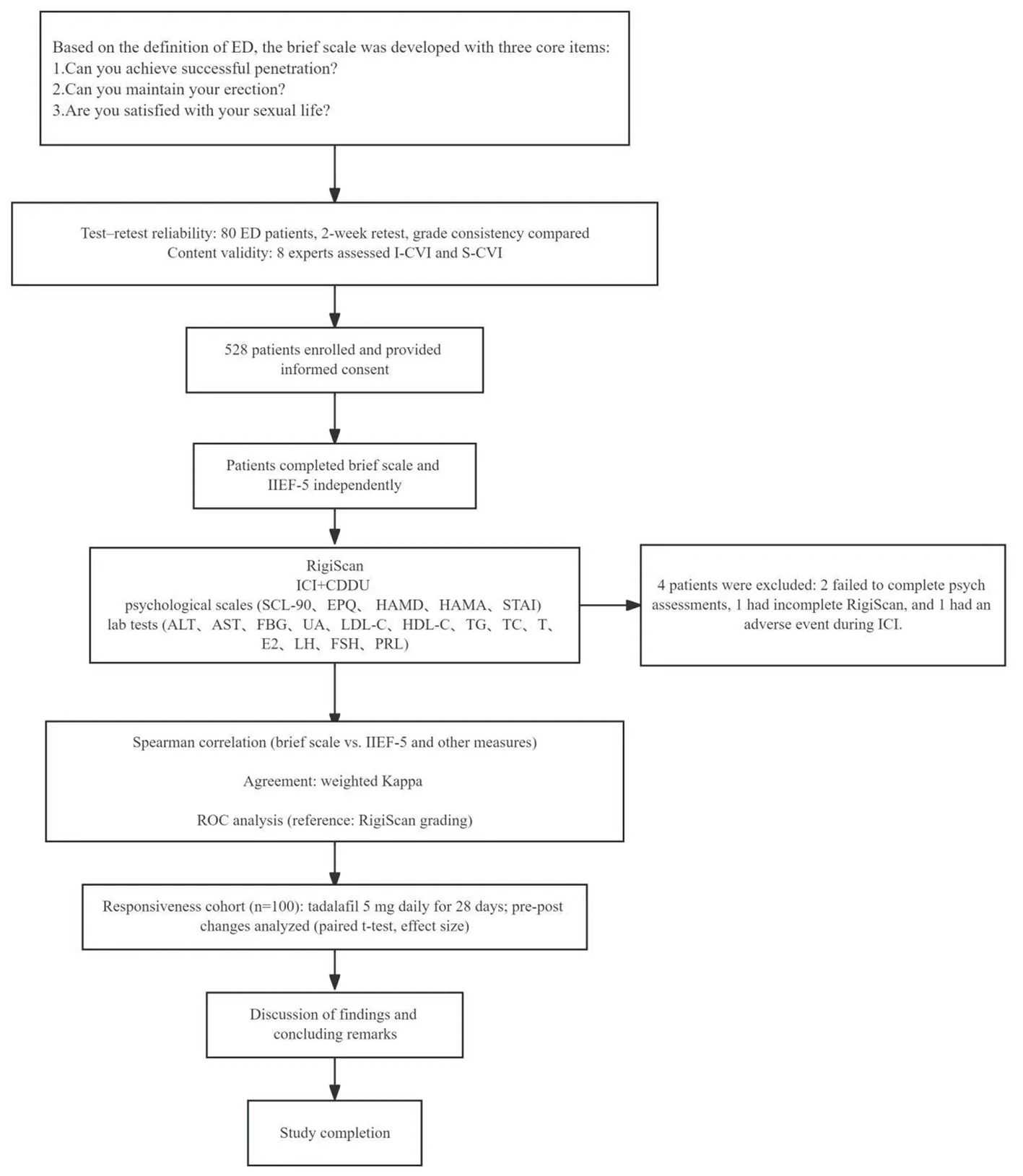
Study design and procedures.

The flowchart is shown in Figure 1. The brief scale developed in this study comprises three items, derived primarily from the widely accepted definition of ED: “the persistent inability to attain or maintain an erection of sufficient rigidity for satisfactory sexual intercourse” (19). From this definition, we extracted three core pathological components: (i) inability to achieve adequate erection (initiation failure), (ii) inability to maintain sufficient rigidity (maintenance failure), and (iii) inability to complete satisfactory sexual intercourse (functional endpoint). These correspond to the following three items: “Can you achieve successful penetration?”, “Can you maintain your erection?”, and “Are you satisfied with your sexual life?” For the first two items, three response options are provided: “almost every time,” “sometimes,” and “almost never.” The third item offers two response options: “almost every time” and “almost never.” The scale employs a branching logic for scoring, with the assessment window set at the preceding 6 months—a timeframe that aligns both with the persistence requirement of the ED definition and with the recall period of the IIEF-5 to ensure comparability between the two instruments. It should be noted that despite the identical recall period, the branching algorithm of the present scale allows patients to complete severity grading even if they have had only a single or infrequent sexual attempt, thereby effectively reducing the risk of missing data due to sexual inactivity. The specific procedure is as follows: if the respondent selects “almost never” on the first item, severe ED is directly assigned; otherwise, the respondent proceeds to the second item. If “almost never” is chosen on the second item, moderate ED is diagnosed; if not, the third item is administered. On the third item, a response of “almost never” yields a classification of mild ED, whereas a response of “almost every time” indicates normal erectile function. In contrast to the IIEF-5 and other existing instruments, the present scale abandons the conventional approach of summing item scores. Instead, it employs a flowchart-based algorithm: depending on the patient's response, the next question is presented or an immediate assessment result is generated.

### 2.2 Scale validation

The feasibility of the novel brief scale was primarily validated by examining its content validity and test–retest reliability (20). For test–retest reliability, 80 patients with ED completed the brief scale on two separate occasions, 2 weeks apart, and the consistency of their severity classifications between the two assessments was compared. Content validity was evaluated by eight experts in relevant fields. The experts assessed each item and the scale as a whole, yielding the item-level content validity index (I-CVI) and the scale-level content validity index (S-CVI), respectively. A four-point rating scale was used to quantify the relevance of each item to the assessment of ED: 1 (not relevant), 2 (somewhat relevant), 3 (quite relevant), and 4 (highly relevant). The indices were calculated as follows:

I-CVI = number of experts who rated the item as 3 or 4 ÷ total number of participating experts.

S-CVI = mean of the I-CVIs across all items (21).

### 2.3 Comparative analysis

This study was conducted from January to October 2025 in the andrology ward and outpatient clinic of Jiangsu Provincial Hospital of Chinese Medicine, and was approved by the hospital's ethics committee (No. YJZ202218). The sample size was determined in accordance with the quality criteria for measurement property studies outlined by the COSMIN (Consensus-based Standards for the selection of health Measurement INstruments) initiative (22). A total of 528 patients were ultimately enrolled. Each participant independently completed both the brief scale and the IIEF-5, and during hospitalization underwent ICI testing, nocturnal RigiScan monitoring, a battery of psychological assessments the Symptom Checklist-90 (SCL-90), the Eysenck Personality Questionnaire (EPQ), the Hamilton Depression Rating Scale (HAMD), the Hamilton Anxiety Rating Scale (HAMA), and the State–Trait Anxiety Inventory (STAI), and a panel of laboratory tests (alanine aminotransferase, aspartate aminotransferase, fasting blood glucose, uric acid, low-density lipoprotein cholesterol, high-density lipoprotein cholesterol, triglycerides, total cholesterol, testosterone, estradiol, luteinizing hormone, follicle-stimulating hormone, and prolactin). Scores from the brief scale and the IIEF-5 were then correlated with the results of each examination. To evaluate test–retest reliability, 80 eligible patients with ED completed the brief scale and repeated the assessment after a 2-week interval. In addition, a separate cohort of 100 patients who met the inclusion criteria was newly enrolled. After completing the brief scale and the IIEF-5, these patients received tadalafil 5 mg once daily. Each treatment course lasted 28 days, with follow-up visits every 14 days. After one course, the patients again completed the brief scale and the IIEF-5, and the changes in scores from baseline were assessed (23).

### 2.4 Statistical analysis

Statistical analyses were performed using IBM SPSS Statistics (version 25.0). Continuous variables were assessed for normality. Data following a normal distribution are presented as mean ± standard deviation (SD), while non-normally distributed data are expressed as median with interquartile range (Q1, Q3). Correlations between variables were evaluated using Spearman's rank correlation coefficients. The strength of correlations was interpreted according to the classification proposed by Mukaka in the Malawi Medical Journal: 0.00–0.10 negligible, 0.10–0.39 weak, 0.40–0.69 moderate, 0.70–0.99 strong, and 1.00 perfect (24). Test–retest reliability was assessed using the intraclass correlation coefficient (ICC). ICC values were categorized as follows: < 0.50 poor reliability, 0.50–0.75 moderate reliability, 0.75–0.90 good reliability, and >0.90 excellent reliability (25). For all analyses, a two-tailed *P*-value < 0.05 was considered statistically significant. To evaluate the concordance between the two scales in severity classification, we calculated weighted Cohen's kappa coefficients (using quadratic weights) and estimated their 95% confidence intervals using the Bootstrap method (1,000 resamples). In addition, receiver operating characteristic (ROC) curve analysis was performed with RigiScan NPTR grading as the reference standard, and the area under the curve (AUC), sensitivity, specificity, positive predictive value (PPV), and negative predictive value (NPV) were computed to evaluate the diagnostic performance of the brief scale for organic ED.

## 3 Results

### 3.1 Validation of the novel brief scale

**Figure 2:**
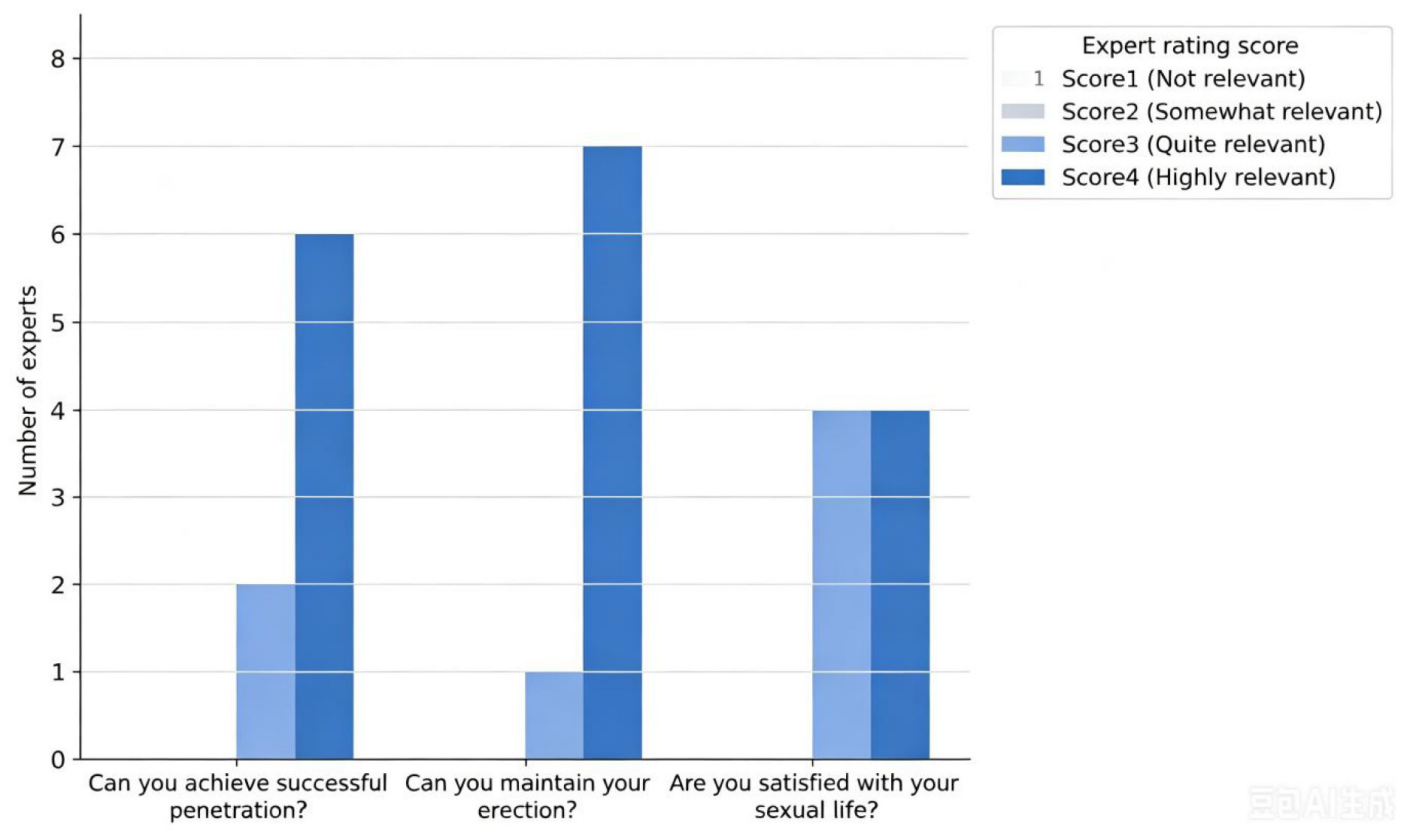
Item-level content validity indices (I-CVIs) for the three items of the brief scale.

All three items achieved an I-CVI of 1.00, exceeding the recommended threshold of 0.78, indicating that each item is highly relevant to the ED construct. The S-CVI was 1.00, demonstrating excellent overall content validity (Figure 2). Test–retest reliability analysis yielded ICCs of 0.991, 0.979, and 0.973 for the individual items, and an ICC of 0.969 for the total score, all meeting the criterion for excellent reliability. An F-test revealed no statistically significant differences between the two measurement occasions for any item or the total score, further confirming the stability of the scale (Table 1).

**Table 1:** Test–retest reliability of the brief scale items.

Item	Sample size (*n*)	Intraclass correlation coefficient (ICC)	*P*-value
Item 1	80	0.991	< 0.001
Item 2	80	0.979	< 0.001
Item 3	80	0.973	< 0.001
Total score	80	0.969	< 0.001

### 3.2 Comparative analysis of the novel brief scale and the IIEF-5

A total of 524 men with ED were included in the final analysis (among the 4 excluded patients: two withdrew due to failure to complete all psychological assessments, one was excluded owing to incomplete RigiScan recording, and 1 did not complete the ICI procedure because of an adverse event during the injection). The distributions of age, disease duration, body mass index (BMI), ALT, AST, fasting blood glucose, uric acid, LDL-C, HDL-C, triglycerides, total cholesterol, T, E_2_, LH, FSH, PRL, total erectile time, number of effective erections, mean tip rigidity, mean tip tumescence, duration of tip rigidity ≥60%, tip rigidity activity units (RAU), tip tumescence activity units (TAU), mean base rigidity, mean base tumescence, duration of base rigidity ≥60%, base RAU, base TAU, total bilateral peak systolic velocity (PSV), total bilateral end-diastolic velocity (EDV), total bilateral resistive index (RI), and the scores on the Symptom Checklist-90 (SCL-90), EPQ, HAMD, and HAMA all deviated significantly from normality. The STAI score was normally distributed (Table 2).

**Table 2:** Baseline demographic and clinical characteristics of the study population (*n* = 524).

Variable	Total (*n* = 524)
Age (years)	35.00 (30.00, 43.00)
Disease duration (months)	12.00 (6.00, 36.00)
BMI (kg/m^2^)	25.06 (22.67, 27.68)
ALT (U/L)	21.50 (14.00, 32.00)
AST (U/L)	20.00 (17.00, 25.00)
Fasting blood glucose (mmol/L)	4.74 (4.47, 5.10)
Uric acid (μmol/L)	370.00 (317.25, 418.75)
LDL-C (mmol/L)	2.84 (2.40, 3.32)
HDL-C (mmol/L)	1.07 (0.92, 1.29)
Triglycerides (mmol/L)	1.40 (0.93, 2.06)
Total cholesterol (mmol/L)	4.42 (3.90, 4.98)
Testosterone (ng/dl)	443.52 (336.96, 568.80)
Estradiol (ng/L)	30.00 (24.00, 37.00)
LH (mIU/dl)	5.50 (4.19, 7.19)
FSH (mIU/dl)	4.33 (3.12, 5.98)
Prolactin (ng/dl)	18.80 (14.34, 24.51)
Hypertension	
0	430 (81.9%)
1	94 (18.1%)
Diabetes	
0	367 (70.0%)
1	157 (30.0%)
Total erectile time (min)	59.63 (28.31, 94.38)
Number of effective erections	2.00 (0.00, 3.00)
Mean tip rigidity (%)	59.00 (48.00, 69.00)
Mean tip tumescence (%)	35.00 (28.00, 42.00)
Duration of tip rigidity ≥60% (min)	31.50 (9.63, 59.50)
Tip RAU	35.00 (15.25, 59.00)
Tip TAU	21.00 (9.00, 35.00)
Mean base rigidity (%)	60.00 (50.00, 70.00)
Mean base tumescence (%)	34.00 (28.00, 39.00)
Duration of base rigidity ≥60% (min)	32.00 (10.00, 59.50)
Base RAU	37.00 (18.00, 60.75)
Base TAU	21.00 (10.00, 34.00)
Total bilateral PSV (cm/s)	53.85 (35.40, 69.60)
Total bilateral EDV (cm/s)	0.90 (0.78, 4.21)
Total bilateral RI	1.94 (1.77, 1.97)
SCL-90 score	55.00 (32.00, 79.75)
EPQ score	38.00 (32.00, 43.00)
HAMD score	13.00 (7.00, 19.00)
HAMA score	14.00 (9.00, 20.00)
STAI (state anxiety subscale)	87.43 ± 18.10

### 3.3 Comparison of the brief scale and the IIEF-5

Of the 528 enrolled patients, 524 completed the study. According to the IIEF-5, 140 were classified as having severe ED, 238 as moderate ED, 141 as mild ED, and 5 as no ED. In comparison, the brief scale classified 152 as severe, 247 as moderate, 120 as mild, and 5 as no ED. Because the severity grades derived from both instruments were not normally distributed, Spearman's rank correlation was employed. The analysis demonstrated a positive correlation between the two grading classifications, with a correlation coefficient of ^*^r^*^ = 0.77 (*P* < 0.001), indicative of a strong correlation (Table 3).

**Table 3:** Correlation between the brief scale and the IIEF-5.

Item	Correlation coefficients (brief scale score)
IIEF-5 score	0.77

**Figure 3:**
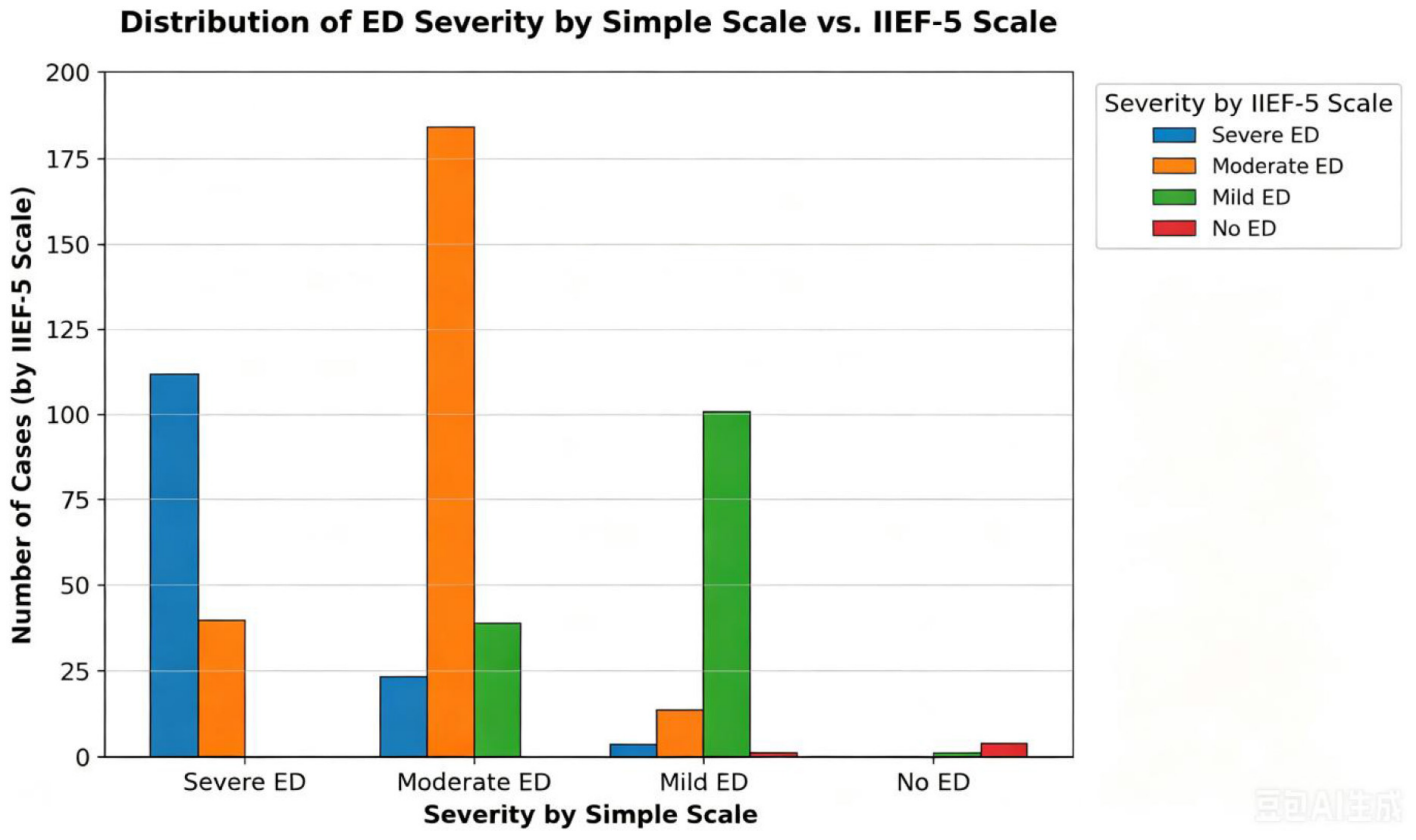
Cross-tabulation of ED severity classifications between the brief scale and the IIEF-5.

To evaluate the agreement between the newly developed brief scale and the validated comparator instrument IIEF-5 in classifying ED severity, a 4 × 4 cross-tabulation was constructed. The two scales yielded identical severity grades in 401 cases, producing an overall agreement rate of 76.5%. The agreement rates for severe, moderate, mild, and no ED were 80.0, 77.3, 71.6, and 80.0%, respectively (Figure 3 and Table 4). The weighted Cohen's Kappa coefficient was 0.773 (95% CI: 0.725–0.817), indicating good-to-excellent classification concordance.

**Table 4:** 4 × 4 cross-tabulation between the brief scale and the IIEF-5.

Severity	Severe ED	Moderate ED	Mild ED	No ED	Total
Severe ED	112	40	0	0	152
Moderate ED	24	184	39	0	247
Mild ED	4	14	101	1	120
No ED	0	0	1	4	5
Total	140	238	141	5	524

**Figure 4:**
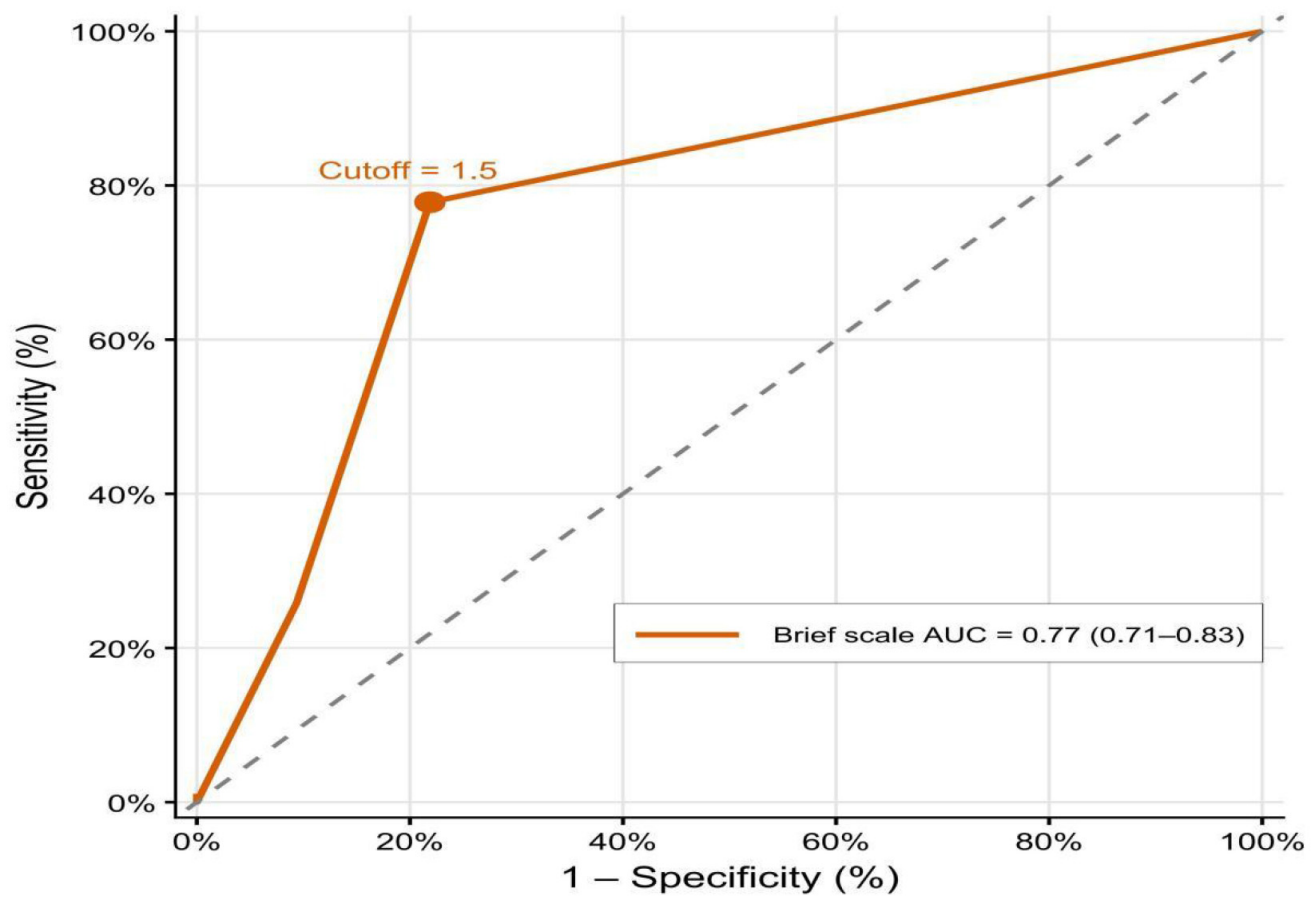
ROC curve of the brief scale for diagnosing ED.

For the 123 patients with discordant classifications, the results of the two scales were further compared with findings from RigiScan NPTR monitoring and ICI testing. When compared against RigiScan grading criteria as the reference standard (26), the brief scale was concordant in 60 cases (25 severe, 31 moderate, 4 mild), whereas the IIEF-5 was concordant in 55 cases (19 severe, 25 moderate, 11 mild). When compared with ICI-derived classifications (27), the brief scale was concordant in 34 cases (14 severe, 14 moderate, six mild), whereas the IIEF-5 was concordant in 46 cases (15 severe, 15 moderate, 16 mild; Tables 5, 6). These findings suggest that the brief scale may offer an advantage in assessing organic ED, particularly moderate-to-severe forms, whereas the IIEF-5 appears superior in evaluating vasculogenic ED. However, it should be noted that the clinical significance of these differences requires further validation. Using RigiScan grading as the reference standard, an ROC curve was constructed for the brief scale in diagnosing organic ED, yielding an AUC of 0.77 (95% CI: 0.71–0.83). At the cut-off point determined by the maximum Youden index, the corresponding sensitivity was 77.8%, specificity 78.1%, false-positive rate 21.9%, false-negative rate 22.2%, positive predictive value 75.3%, and negative predictive value 80.4%. These results indicate that the scale has moderate discriminatory ability for organic ED (Figure 4).

**Table 5:** Concordance of the brief scale and IIEF-5 with RigiScan-derived severity grades.

Item	Severe ED	Moderate ED	Mild ED	No ED	Total
Number of concordant cases (brief scale)	25	31	4	0	60
Number of concordant cases (IIEF-5)	19	25	11	0	55

**Table 6:** Concordance of the brief scale and IIEF-5 with ICI-derived severity grades.

Item	Severe ED	Moderate ED	Mild ED	No ED	Total
Number of concordant cases (brief scale)	14	14	6	0	34
Number of concordant cases (IIEF-5)	15	15	16	0	46

### 3.4 Associations of the brief scale and the IIEF-5 with RigiScan parameters

The scale classifications and RigiScan variables of the 524 patients were analyzed. Spearman correlation analyses revealed that the brief scale was positively correlated with all RigiScan parameters. Specifically, moderate positive correlations were found for base RAU, total erectile time, base TAU, tip RAU, tip TAU, duration of tip rigidity ≥60%, duration of base rigidity ≥60%, and the number of effective erections; weak positive correlations were observed for mean base tumescence, mean tip tumescence, mean tip rigidity, and mean base rigidity. The IIEF-5 was likewise positively correlated with all RigiScan indices. Of these, strong positive correlations were evident for total erectile time, base TAU, base RAU, tip TAU, tip RAU, duration of tip rigidity ≥60%, and duration of base rigidity ≥60%; moderate correlations were found for the number of effective erections and mean base tumescence; and weak correlations for mean tip tumescence, mean tip rigidity, and mean base rigidity (Table 7).

**Table 7:** Correlation coefficients of the brief scale and IIEF-5 with RigiScan parameters.

Parameter	IIEF-5	Brief scale
Total erectile time (min)	0.637	0.463
Number of effective erections	0.391	0.381
Mean tip rigidity (%)	0.19	0.208
Mean tip tumescence (%)	0.294	0.232
Duration of tip rigidity ≥60% (min)	0.522	0.426
Tip RAU	0.562	0.442
Tip TAU	0.589	0.431
Mean base rigidity (%)	0.151	0.162
Mean base tumescence (%)	0.367	0.292
Duration of base rigidity ≥60% (min)	0.506	0.411
Base RAU	0.598	0.467
Base TAU	0.623	0.457

### 3.5 Associations of the brief scale and IIEF-5 scores with ICI parameters

Analyses of the scale classifications and ICI parameters demonstrated that the brief scale was correlated with all ICI indices, and the direction of these correlations was consistent with vascular physiology: positive correlations were observed with total bilateral PSV, negative correlations with total bilateral EDV, and positive correlations with total bilateral RI; all were weak correlations. The IIEF-5 exhibited an identical pattern of associations, showing significant, albeit weak, positive correlations with bilateral PSV and RI, and a significant weak negative correlation with bilateral EDV (Table 8).

**Table 8:** Correlation coefficients of the brief scale and IIEF-5 with ICI parameters.

Item	Total bilateral PSV (cm/s)	Total bilateral EDV (cm/s)	Total bilateral RI (cm/s)
IIEF-5	0.175	−0.133	0.163
Brief scale	0.159	−0.13	0.165

### 3.6 Associations of the brief scale and IIEF-5 scores with psychological assessments

The associations between the severity classifications of the two scales and the scores on the psychological inventories were examined. The brief scale was negatively correlated with all psychological measures: the correlation coefficients with the SCL-90, EPQ, HAMD, HAMA, and STAI were all weak. Similarly, the IIEF-5 showed negative correlations with each of these psychological scores, and the corresponding correlation coefficients also fell within the weak range (Table 9).

**Table 9:** Correlation coefficients of the brief scale and IIEF-5 with psychological assessment scores.

Item	SCL-90 score	EPQ score	HAMD score	HAMA score	STAI score
IIEF-5	−0.21	−0.228	−0.2	−0.217	−0.278
Brief scale	−0.18	−0.287	−0.216	−0.187	−0.198

### 3.7 Associations of the brief scale and IIEF-5 scores with laboratory parameters

The scale classifications were further examined in relation to the results of laboratory tests. The brief scale exhibited a weak negative correlation with LDL; no significant correlations were found with HDL, T, ALT, AST, fasting blood glucose (FBG), UA, TG, TC, E_2_, LH, FSH, or PRL. The IIEF-5 demonstrated a weak positive correlation with T, whereas its correlations with all other laboratory parameters did not reach statistical significance (Table 10).

**Table 10:** Correlation coefficients of the brief scale and IIEF-5 with laboratory parameters.

Variable	IIEF-5	Brief scale
ALT (U/L)	−0.05	−0.085
AST (U/L)	−0.009	−0.033
Fasting blood glucose (mmol/L)	0.042	−0.013
Uric acid (μmol/L)	−0.036	−0.051
LDL-C (mmol/L)	−0.085	−0.108
HDL-C (mmol/L)	0.078	0.089
Triglycerides (mmol/L)	−0.029	−0.045
Total cholesterol (mmol/L)	−0.072	−0.083
Testosterone (ng/ml)	0.104	0.095
Estradiol (pg/ml)	0.004	0.006
LH (mIU/L)	−0.026	−0.011
FSH (mIU/L)	0.006	−0.032
Prolactin (ng/ml)	−0.004	−0.016

### 3.8 Comparison of changes in the brief scale and IIEF-5 scores before and after treatment

ICCs were computed to assess the agreement between pre- and post-treatment grades for each scale. The ICC was 0.712 for the IIEF-5 and 0.752 for the brief scale, both falling within the moderate-to-good range. When the change scores (post-treatment minus pre-treatment) derived from the two scales were compared, the ICC was 0.659. These findings indicate that both instruments show acceptable repeat-measure consistency over time, and that the change scores exhibit a moderate level of agreement, suggesting that the brief scale captures treatment-related improvements in a pattern similar to that of the IIEF-5 (Table 11). Furthermore, paired *t*-tests revealed that the IIEF-5 grade improved from 1.87 ± 0.75 before treatment to 2.66 ± 0.62 after treatment, and the brief scale grade improved from 2.03 ± 0.82 to 2.76 ± 0.73, with both changes being statistically significant (*P* < 0.001). The effect sizes (Cohen's ^*^d^*^) were 1.65 for the IIEF5 and 1.64 for the brief scale, indicating large responsiveness.

**Table 11:** Comparison of pre- and post-treatment scores for the brief scale and IIEF-5.

Item	Sample size (*n*)	ICC
IIEF-5 (pre vs. post)	100	0.712
Brief scale (pre vs. post)	100	0.752
Difference score between the two scales	100	0.659

## 4 Discussion

Based on the core definition of ED, this study developed a flowchart-based brief assessment tool comprising only three items and systematically evaluated its clinical performance. The mean completion time was 0.9 ± 0.3 min for the severe ED group, 1.4 ± 0.4 min for the moderate ED group, 1.7 ± 0.5 min for the mild ED group, and 1.3 ± 0.5 min overall. The scale demonstrated excellent content validity and test–retest reliability, with a Spearman correlation coefficient of 0.77, an overall agreement rate of 76.5%, and a weighted kappa of 0.773 when compared with the widely used IIEF-5 for severity grading. Discordant cases were predominantly distributed across adjacent severity grades, which is consistent with the clinical reality that ED severity exists along a continuum with inherently overlapping boundaries. These findings indicate that the brief scale possesses good criterion-related validity and can effectively capture the level of erectile function.

ROC analysis showed that the scale achieved an AUC of 0.77 for diagnosing organic ED, with a sensitivity of 77.8% and specificity of 78.1%, indicating moderate discriminatory ability between organic and non-organic ED. In correlation analyses with objective examinations, the brief scale demonstrated consistently positive associations with all RigiScan NPTR parameters, and its correlations with the vascular indices derived from ICI testing were entirely congruent with physiological expectations. However, the correlation coefficients were mostly weak-to-moderate, suggesting that the scale has limited capacity to replace objective vascular function assessment. Notably, further analysis of the discordant cases suggested that the brief scale agreed more closely with RigiScan parameters, whereas the IIEF-5 was more consistent with ICI outcomes. RigiScan recording captures erectile activity occurring during natural sleep and is thus relatively free from subjective psychological influences, making it more indicative of organic erectile capacity (28). Although ICI involves direct pharmacological action, the procedure-related visit anxiety and psychological expectations may still introduce interference (29). The three items of the brief scale focus exclusively on the initiation, maintenance, and completion of erection, with minimal involvement of subjective experiential dimensions such as sexual desire or intercourse satisfaction. This narrow focus may partly explain its closer relationship with RigiScan. Nevertheless, given the overall weak correlations, the clinical significance of this difference remains unclear. Neither questionnaire should be considered a substitute for objective diagnostic tests; the brief scale may only serve as an auxiliary tool for preliminary screening, and its predictive value for organic ED should be interpreted with caution, with the ROC results requiring validation in independent external populations.

In terms of psychological status, the negative correlations observed between the brief scale and scores of psychological distress—including depression and anxiety—align with the clinical consensus that ED and psychological distress often exhibit a bidirectional, reciprocal relationship (30). However, the correlation coefficients were weak, indicating that the scale cannot replace professional psychological assessment instruments. With respect to metabolic and endocrine indices, the brief scale showed a weak negative correlation with LDL, and the IIEF-5 exhibited a weak positive correlation with testosterone. These findings merely suggest a statistical association between questionnaire scores and certain metabolic parameters, without carrying clinically meaningful predictive or diagnostic value. The inference of ED as an early marker of cardiovascular disease still relies on objective metabolic and vascular examinations rather than questionnaire scores alone. Overall, both scales demonstrate limited ability to reflect objective pathophysiological status; their primary clinical value lies in the rapid acquisition of patient-reported outcomes rather than in replacing laboratory or instrumental examinations. Therefore, given the inherent limitations of subjective scales such as the IIEF-5, exploring more objective and precise assessment approaches has become a research hotspot in this field. For instance, Ozlu et al. (31) demonstrated that penile shear wave elastography, as a non-invasive objective examination, can effectively distinguish between different etiologies of ED. This underscores the importance of developing novel, multidimensional assessment tools for the precise diagnosis and management of ED.

Several limitations of the present study should be acknowledged. First, the brief scale remains focused on the unidimensional construct of erection and therefore cannot discriminate among different etiologies of ED; it also does not capture critical information such as the use of erectile aids. Second, the sample was drawn from a single center, and although 524 patients were included, the generalizability of the findings awaits confirmation in multicentre investigations. Third, the study did not perform stratified analyses according to educational attainment or the presence of comorbid pre-mature ejaculation, factors that may influence self-reported outcomes. The test–retest reliability interval was set at 2 weeks; the responsiveness analysis was based on only 100 patients (as an independent cohort) and a single treatment regimen (tadalafil 5 mg once daily). The sensitivity of the scale to other treatment modalities therefore requires further examination. In addition, although the 6-month recall window adopted in this study is consistent with the diagnostic criterion of “persistence” for ED and covers contextual factors such as partner changes and work-related stress, erectile function itself is susceptible to short-term fluctuations (e.g., fatigue on a given night, mood, or specific partner dynamics). The present scale did not distinguish between a single attempt and the average state across multiple attempts, which constitutes a limitation. Future iterations may consider incorporating a separate “current state” item to adjust for recall bias. Furthermore, the relatively wide confidence interval of the AUC from the ROC analysis reflects limited precision in the estimation of diagnostic performance; larger sample sizes and validation of optimal cut-off values in multicentre populations are warranted. Future studies should establish the measurement properties of the scale across diverse populations and clinical settings, determine its minimal clinically important difference, and explore the optimal pathway that integrates patient self-report with objective assessments.

In summary, the novel brief ED scale developed in this study demonstrates sound reliability and validity, requires minimal administration time, and is well accepted by patients. While maintaining a high level of concordance with the IIEF-5, it shows certain advantages in associations with specific objective parameters. Therefore, this scale holds promise as a complementary rapid screening tool for the preliminary assessment of ED, and is particularly well-suited for outpatient screening and large-scale epidemiological surveys.

## 5 Conclusion

In conclusion, this novel brief scale demonstrates potential clinical utility for the rapid assessment of erectile dysfunction severity, offering a time-efficient and convenient alternative that may serve as a complementary rapid screening tool in appropriate clinical contexts.

## Data Availability

The original contributions presented in the study are included in the article/Supplementary material, further inquiries can be directed to the corresponding author.
