# Supplementary material for: Clinical performance of a novel brief erectile dysfunction scale and comparison with the international index of erectile function-5
Source: Front Public Health. 2026 Aug 13;14:1901986. doi: 10.3389/fpubh.2026.1901986 (PMC13518213; doi:10.3389/fpubh.2026.1901986)
Supplement: Supplementary file 1 [file Table_1.docx]

**S1:Brief Erectile Dysfunction Scale**

**Brief Erectile Dysfunction Scale**

**Please answer the following questions based on your actual sexual experience over the last 6 months.**

**Name:  Age:**

**Marital status:
Unmarried ( )   Married ( ) for _____ years**

**Past medical history: Diabetes ( ) Hypertension ( )**

| **Question1. Can you achieve successful penetration?** | | | | |
| --- | --- | --- | --- | --- |
| **□A.Almost every time** | | **□B.Sometimes** | | **□C.Almost never** |
| **Question2. Can you maintain your erection?** | | | |  |
| **□A.Almost every time** | **□B.Sometimes** | | **□C.Almost never** |  |
| **Question3. Are you satisfied with your sexual life?** | | |  |  |
| **□A.Almost every time** | **□B.Almost never** | |  |  |
| **Normal** | **Normal** | | **Moderate** | **Severe** |

**Surgery history ( )  Trauma history ( ) Masturbation history ( )**

**Smoking and alcohol history: Smoking ( )  Drinking ( )**
